# Social and Poor-Law Problems

**Published:** 1908-03-07

**Authors:** 


### SOCIAL AND POOR-LAW PROBLEMS.

### "SOCIALISM AND THE DOCTOR."

Under this title Dr. Lawson Dodd gave an interesting lecture at St. Anne's Hall, Clapham. He pointed out that medicine had gone through three stages of development—the mysterious one, when the physician was the priest or the witch; the guild or trade-union one of the later Middle Ages; and only in the latter part of the nineteenth century had it entered the professional stage. Dr. Dodd dated this change from the Medical Act of 1858. This Act was, in Dr. Dodd's interpretation of the word, a "Socialistic" one, in that it protected the public by fixing a standard of education and qualification for medical men, keeping a register of those legally qualified, and acting also as a medical court of justice. In this connection Dr. Dodd justified the much-maligned "medical etiquette," which forbade advertising and struck off the Register practitioners who, after warning, continued to indulge in it. For, though some people justified quackery as breaking the phalanx of professionalism, there was nothing more detrimental to social welfare than the quack, with his secret remedies, exorbitant charges, and unscrupulous advertising; and he believes that when it comes, Socialism will have none of such quackery.

Scientific medicine, on the other hand, has an essentially democratic basis. Its tendency is to push men in the direction of advanced social reform. As the first step towards improving the medical profession was State interference, as exemplified in the Act of 1858, the next was State organisation. In proof of the latter point Dr. Dodd cited the number of medical men who were already directly employed by the State. There are now between three and four thousand medical men employed in the Army and Navy Medical Service, and, if Mr. Haldane's scheme is carried out, a still larger number of medical men will be enlisted. There are also the doctors employed in the Colonial service. To these should be added the medical officers of health, the prison surgeons and doctors working under the Factory Acts, the large staff of the Metropolitan Asylums Board, the staff of asylums, and (a late addition) the staff of school doctors. These services form the foundation of what will develop into a great edifice, and each will absorb more and more medical practitioners. Besides these, there are many medical practitioners who are partly paid by the State, such as the Poor-law doctors, those employed by the Post Office, etc. These public posts are popular, and good work is done by them; and Dr. Dodd gave a flat denial to the notion that the possession of public offices makes men lazy.

The existence of these posts, and the good done through their existence, prove, Dr. Dodd said, that in recommending the State organisation of the medical profession he is not putting forward any revolutionary plan. In opposition to the doctor employed by the State, he drew a picture of the story of the average medical man—the general practitioner who, well trained and qualified, must as a rule start his career by a period of enforced idleness, and, when he begins to get patients, knows that each one he wins is taken from a rival practitioner, who is probably older than he, and perhaps more worn with the trials of life. Moreover, he finds out that the qualifications that attract patients to him are not his professional ones, but rather matters of manners, breeding, and sociability. Then, realising the sort of people he has to do with, he is tempted to be civil rather than wholly sincere, to prescribe pleasant remedies rather than rough ones, in order to keep his patients' favour. He has also other competitors to contend with, less legitimate than rival practitioners—the prescribing chemist, hospitals and all kinds of societies which give free treatment, the quack, and the man who is not "playing the game." The result is that in many cases it is impossible for a man to make a decent livelihood. The average income of a doctor is at present about £250 a year—not much more than the wage of a good mechanic, and a very small reward for the time and cost of a medical education. In some places the price of a consultation and medicine is a shilling, in others only sixpence! And Dr. Dodd mentioned that even trades-unions were not above sweating their doctors, and gave instances to show how, when the medical men in a certain town had united to refuse the terms offered by a union, the officials of the latter imported a raw youth who was willing to accept their practice on a basis of 10d. per head per annum, or something less than a farthing a visit. Under sane conditions such things would not exist, any more than it would be permitted to a medical man to work day and night, as many unfortunately do at present.

Dr. Dodd pointed out that the middle class are the chief sufferers from the present chaotic system. The really poor are taken to a hospital, where they have every advantage that modern knowledge can give, while the lower middle class, treated at home, suffer discomforts which must retard recovery and even endanger life. Withal, the mere fact that the patient "employs" a doctor puts the latter in a position which militates against his efficiency, because it hinders him from speaking plain truths and saves the patient's feelings at the expense of his recovery. The remedy is to make the whole medical profession a civil service—a public service. It is desirable to add to the numbers of medical officers of health, and to get rid of the "half-time" M.O.H.—the man who gets some £30 a year for his public health work, and has to earn the rest of his living from people whose interests are often entirely opposed to it. It is desirable to make the school medical staff larger, so that the eyes, ears, throat, &c., of every child should be regularly and systematically attended to.

There should be an increased medical staff for our prisons, for there were many there who should by rights be in hospitals or asylums; and, conversely, there were many in asylums who might benefit by the severer discipline which they would receive elsewhere. He would like to see the Poor-law medical service transferred to the jurisdiction of the public health authority, with the medical officer of health as chief. He desired all hospitals to be nationalised, controlled by the community, and responsible to the community, and would like also to see a wider notification of disease—in fact, that all disease should be notified, so that we might know exactly how much disease there was in a community, and the proportion of disease to death. For, as he pointed out, even when a patient recovers, he bears the scars of his illness in weakness of some kind, and the whole trend of modern science is to show that disease is in any true sense less curable than was formerly thought, but much more preventible, and the medical profession of the future will therefore devote itself more to prevention than of old; and this preventive service, which works for the good of the community as a whole, should be arranged for, and controlled by the community. In fact, the keynote of medical progress now is organisation in the great army that fights against disease and death.

The lecture was attentively listened to by a large audience, and at the end many questions were asked, to which Dr. Dodd gave clear and pertinent answers.

